# Diagnostic benefits of platelet-to-lymphocyte, neutrophil-to-lymphocyte, and albumin-to-globulin ratios in dogs with nasal cavity diseases

**DOI:** 10.1186/s12917-024-03876-5

**Published:** 2024-02-03

**Authors:** Sarah Rösch, Julia Woitas, Stephan Neumann, Michaele Alef, Ingmar Kiefer, Gerhard Oechtering

**Affiliations:** 1 Small Animal Department, ENT-Unit, University of Leipzig, An den Tierkliniken 23, Leipzig, SN DE- 04103 Germany; 2 Current affiliation: Clinic for Small Animals, University of Veterinary Medicine Hannover Foundation, Bünteweg 9, Hannover, NI DE-30559 Germany; 3 Small Animal Department, University of Leipzig, An den Tierkliniken 23, Leipzig, SN DE-04103 Germany; 4 Institute of Veterinary Medicine, University of Goettingen, Burckhardtweg 2, Goettingen, NI DE-37077 Germany

**Keywords:** Nasal tumor, Sinonasal aspergillosis, Lymphoplasmacytic rhinitis, Rhinoscopy

## Abstract

**Background:**

A multimodal approach for diagnostic tests under anesthesia is required to diagnose nasal cavity pathology (NP) reliably in dogs. Blood test results may provide clues to the suspected NP.

**Methods:**

This prospective blinded study assessed 72 dogs with chronic nasal discharge due to NPs, and 10 healthy dogs as the control group (CG). NPs were diagnosed using whole-body computed tomography (CT), upper airway endoscopy, examination of nasal mucosal swabs by bacterial and fungal culture, and histopathological examination of nasal mucosa biopsies. The exclusion criteria were the presence of any additional diseases or corticosteroid pre-treatment. In consideration of these exclusion criteria, 55 dogs entered the study. Dogs were classified into benign (benign tumors, idiopathic rhinitis (IR), and others) and malignant (carcinomas and sarcomas) NP groups. Blood count and blood chemistry tests were performed. The neutrophil-to-lymphocyte ratio (NLR), platelet-to-lymphocyte ratio (PLR), and albumin-to-globulin ratio (AGR) were calculated and compared.

**Results:**

25 dogs with malignant NP (13 and 12 with carcinomas and sarcomas, respectively) and 30 dogs with benign NP (seven with benign tumors,13 with IR, and 10 others) were included. In general, in dogs with NP there were only slight abnormalities in complete blood count. However, PLR was significantly higher in dogs with malignant NP (carcinoma and sarcoma) than in those with benign NP and in the CG. Compared with the CG, the NLR was significantly increased in all dogs with NP, and the AGR was mild but significantly lower, except in dogs with sarcomas and benign tumors.

**Conclusions:**

In dogs with nasal disease alone, there are usually no marked abnormalities in blood count. However, while mildly increased NLR and decreased AGR can be observed in almost all NPs, an increased PLR may indicate a malignant NP and can be used as an additional screening tool in dogs with nasal discharge due to nasal cavity pathology.

## Background

Nasal cavity pathologies (NPs) are more often responsible for chronic nasal discharge (ND) than systemic diseases in dogs [1]. NPs include nasal neoplasms (NN; much more commonly malignant than benign [2]), oronasal defects (ONDs [3]), mycotic rhinitis (such as sinonasal aspergillosis (SNA)), nasal foreign bodies (FBs), nasal entrance pathologies, and idiopathic rhinitis (IR) [1, 3, 4]. Furthermore, parasitic diseases, e.g., Pneumonyssoides caninum, have been described [4].

The diagnosis of NPs is best made with the aid of a combination of tests, specifically computed tomography (CT), rhinoscopy, and histopathological examination of biopsies of endoscopically visualized tumor tissue or nasal mucosa, as well as nasal swabs for mycological and/or microbiological examination by culture [1, 3, 5]. Furthermore, cross-sectional imaging can evaluate possible dental disease, frontal sinus involvement, the integrity of the cribriform plate, and the anatomic extent of neoplastic disease, which is of prognostic and therapeutic importance [5, 6].

Idiopathic rhinitis (IR) is a special type of benign NP. It is a benign inflammation of the nasal mucosa without an identified etiology and is among the most common causes of ND in dogs after malignant neoplasia or ONDs. However, IR treatment is often difficult because its etiology has not been conclusively determined [7]. Furthermore, conventional antibiotic therapy usually does not result in a cure for IR [8]. Therapies for IR include administering corticosteroids, ciclosporin, or desensitization [7, 9]. A pilot study described different treatment protocols with meloxicam and/or corticosteroids [10]. Types of mucosal inflammation found in dogs with IR, including lymphoplasmacytic inflammation, can also be found in dogs with ONDs or nasal FBs [3]. Therefore, IR diagnosis cannot be based solely on histopathologic examination results [3]. Instead, IR diagnosis should be a diagnosis by exclusion.

Comprehensive and costly diagnostic procedures in dogs with chronic ND must be performed under anesthesia. Hence, whether blood values differ in dogs with the NPs mentioned above and whether they can be used instead of cost-intensive methods in anesthesia to diagnose nasal cavity disease requires elucidation. Furthermore, blood markers in course of treatment for benign nasal cavity diseases are needed, to avoid repeated examinations under anesthesia. In contrast to dogs with systemic causes of ND, such as leishmaniasis or ehrlichiosis [11], previously published isolated reports of dogs with NPs indicated that only minor abnormalities in blood count or blood chemistry were present in the few dogs that underwent blood tests [12]. However, it is unclear if the dogs of these few studies were otherwise healthy or suffered of diseases that can influence blood values. Blood parameter ratios other than single blood values have not been reported to date and could differ even more significantly between dogs with nasal diseases and healthy dogs or dogs with systemic diseases, thereby making differentiation of nasal disease easier for clinicians.

Formation of blood parameter ratios leads to results that are less susceptible to interference than results from individual parameters [13]. Hence, different parameters can be used for this purpose, such as the number of certain leukocyte fractions or the negative acute-phase protein albumin. The neutrophil-to-lymphocyte ratio (NLR) and albumin-to-globulin ratio (AGR) have been evaluated as diagnostic markers in dogs with malignant and benign soft tissue tumors [13]. AGR serves as a prognostic tumor marker in human medicine. However, to the best of the authors’ knowledge, AGR in veterinary medicine has only been studied in dogs with leishmaniasis [14, 15] and soft tissue tumors [16]. In contrast, NLR has been studied in dogs with mast cell tumors, lymphoma, periodontitis, and sarcoma [13, 16–18]. Thus, NLR has been reported to be significantly increased and AGR to be decreased in canine malignant tumors [13]. Hence, how these ratios behave in dogs with nasal diseases and if the AGR may be comparably low in dogs with systemic disease leading to nasal discharge as in leishmaniasis, remains unclear.

Although thrombocytosis can be associated with various neoplastic, metabolic, and inflammatory processes [19] and is used as a prognostic marker in many neoplastic diseases in human medicine [20], the platelet-to-lymphocyte ratio (PLR) in dogs has rarely been studied [21–23]. Given increases in PLR in neoplastic diseases in human medicine, whether PLR could be an indicator for malignant nasal tumors in dogs is of clinical interest.

Therefore, this study aimed to evaluate blood count and NLR, AGR, and PLR for diagnostic utility in a group of dogs with NPs. We compared these ratios between dogs of the CG and dogs with benign and malignant NPs and among both diseased groups and performed subsequently subgroup and supergroup comparisons. We hypothesized that blood ratios, rather than the blood count itself would differ between dogs with malignant and benign NPs.

## Results

Of the 72 dogs assessed, 55 were included in this study. Based on the findings of CT, endoscopy, histological, bacteriological, and mycological examination findings, 25 of the 55 dogs (45%; CI_95%_ 33–59) were assigned to the malignant NN group [carcinomas (*n* = 13) and sarcomas (*n* = 12)], and thirty (55%; CI_95%_ 42–67) were assigned to the benign pathology group [benign NN (*n* = 7), IR (*n* = 13), and others (*n* = 10)]. In addition, the results for blood values and blood ratios were compared to those of the 10 control dogs. The median blood parameter values are shown in Table 1.

**Table 1:** Blood parameters of dogs with nasal discharge compared with those in the control group (CG). Indicated are the non-parametric data with median and interquartile range [in square brackets]; parametric data are illustrated with mean ± standard deviation.

		Dogs with nasal cavity pathology and nasal discharge					CG
	Reference range	Carcinomas	Sarcomas	Benign tumors	IR	Others	Healthy
Hematocrit (l/l)	0.44–0.52	0.462 ± 0.086	0.532 ± 0.068	0.524 ± 0.051	0.46 ± 0.073	0.489 ± 0.04	0.499 ± 0.033
Leukocytes (G/l)	6–12	9.5 [8.45–17.4] ***p*** **= 0.0032**	10.2 [8.58–12] ***p*** **= 0.0231**	9.2 [8.6–9.7]	13.55 [10.43–17.68] ***p*** **<0.0001**	12.55 [8.5–14.63] ***p*** **= 0.0051**	7 [5.95–8.23]
Segmental Neutrophils (G/l)	3–9	6.8 [5.8–13.8] ***p*** **= 0.0007**	7.8 [5.6–8.8] ***p*** **= 0.0081**	6.7 [5.6–7.7]	10.9 [8–13.8] ***p*** **<0.0001**	8.3 [6–10.9] ***p*** **= 0.0047**	4.2 [3.8–4.9]
Lymphocytes (G/l)	1–3.6	1.8 [1.4–2.5]	1.7 [1.3–2.3]	2.1 [1–2.4]	2 [1.8–2.6]	2.1 [1.6–3]	2.1 [1.9–2.9]
Eosinophils (G/l)	0.04–0.6	0.4 [0.2–0.6]	0.6 [0.4–0.8]	0.5 [0.2–0.8]	0.4 [0.1–0.8]	0.7 [0.3–0.8]	0.2 [0.2–0.3]
Monocytes (G/l)	0.04–0.5	0.7 [0.3–1] ***p*** **= 0.0016**	0.3 [0.2–0.6]	0.3 [0.2–0.3]	0.5 [0.3–0.6] ***p*** **= 0.0269**	0.4 [0.2–1.1]	0.2 [0.2–0.3]
Platelets (G/l)	150–500	300 [218–483.5]	317.5 [261.5–473]	246 [170–289]	299.5 [257–371.3]	240.5 [203.5–288]	241.5 [209.5–320.3]
NLR		6.2 [3.06–7.23] ***p*<0.0001**	4.82 [3.85–5.07] ***p*<0.0001**	3.29 [2.43–6.78] ***p* = 0.0031**	4.22 [3.84–6.68] ***p*<0.0001**	3.89 [2.63–5.53] ***p* = 0.0008**	1.95 [1.71–2.32]
PLR		166.7 [102.1–273] ***p* = 0.0493**	185.2 [131.2–262.7] ***p* = 0.0071**	125.7 [71.25–188.9]	134.2 [120.5–195.9]	106.9 [89.67–203.2]	123.5 [95.84–143.9]
Albumin (g/l)	25–44	38.05 ± 3.75	41.82 ± 4.85	40 ± 2.7	38.83 ± 2.93	39.09 ± 3.91	38.92 ± 1.91
Globulin (g/l)	< 45	29.1 [22.9–33.9] ***p*** **= 0.0008**	26 [22.15–27.9] ***p*** **= 0.0310**	24 [20.7–24.2]	25.6 [23.2–28.6] ***p*** **= 0.0241**	24.2 [22.6–29.2]	19 [16.9–23.3]
AGR		1.43 ± 0.46 ***p* = 0.0063**	1.69 ± 0.5	1.74 ± 0.12	1.51 ± 0.22 ***p* = 0.0014**	1.58 ± 0.31 ***p* = 0.0213**	1.98 ± 0.39

Significant differences compared to the control group are indicated with *p*-values in the respective columns

NLR, Neutrophil-to-lymphocyte ratio; PLR, Platelet-to-lymphocyte ratio; AGR, Albumin-to-globulin ratio

### Group-specific characteristics

Malignant neoplastic NPs (malignant NN 25/55; 45%; CI_95%_ 33–59). Carcinoma was diagnosed in 13 of the 25 dogs (52%; CI_95%_ 34–70) diagnosed with malignant NPs (adenocarcinomas, *n* = 4; low differentiated carcinomas, *n* = 3; transitional cell carcinomas, *n* = 3; carcinoma in situ, *n* = 1; carcinoma, *n* = 1; squamous cell carcinoma, *n* = 1). On the other hand, 12 of the 25 dogs (48%; CI_95%_ 30–67) were diagnosed with sarcoma (chondrosarcomas, *n* = 7; sarcomas, *n* = 3; osteosarcoma, *n* = 3; hemangiosarcoma, *n* = 1). Twenty-one (84%; CI_95%_ 65–94) and four (16%; CI_95%_ 6–35) of the 25 dogs were normocephalic, and brachycephalic, respectively. Sixteen and nine of the 25 dogs were male [64%; CI_95%_ 45–80; intact (*n* = 8) and neutered (*n* = 8)], and female [36% CI_95%_ 20–55; intact (*n* = 4) and neutered (*n* = 5)], respectively.

Benign tumors (7/55; 13%; CI_95%_ 6–24). Benign masses were diagnosed in seven dogs, including nasal polyp (*n* = 1), hamartomas (*n* = 2), benign hyperplasia (*n* = 2), chronic inflammatory growth (*n* = 1), and polypous rhinitis (*n* = 1). Six (86%; CI_95%_ 49–99) and one (14%; CI_95%_ 1–51) of the seven dogs were normocephalic and brachycephalic, respectively. Three and four of the seven dogs were male [43%; CI_95%_ 15–75; intact (*n* = 2) and neutered (*n* = 1)] and female [57%; CI_95%_ 25–84; intact (*n* = 1) and neutered (*n* = 3)], respectively.

IR was diagnosed in 13 of the 55 dogs (24%; CI_95%_ 14–36). Hematology (NLR and PLR) could only be performed in 12 of these 13 dogs, and blood chemistry (including AGR) was performed in all 13 dogs. All the dogs were normocephalic (100%; CI_95%_ 77–100). Histology revealed bilateral lymphoplasmacytic rhinitis (4/13), chronic rhinitis (5/13) and neutrophilic rhinitis (4/13). Eight and five of the 13 dogs were male [62%; CI_95%_ 36–82; intact (*n* = 3) and neutered (*n* = 5)) and female (38%; CI_95%_ 18–65; intact (*n* = 2) and neutered (*n* = 3)], respectively.

Other benign inflammatory nasal cavity pathologies. Ten of the 55 dogs (18%; CI_95%_ 10–30) were diagnosed with other NPs, resulting in ND. These included primary SNA (*n* = 1; histology: necrotizing rhinitis) or SNA caused by a foreign body (*n* = 2; histology: neutrophilic and mycotic rhinitis), rhinitis after diagnosis and resolution of SNA (histology: chronic rhinitis) in which mycological examinations were negative (*n* = 1), dental root pathology (*n* = 2; histology: neutrophilic and chronic rhinitis), nasal or nasopharyngeal FB (*n* = 2), rhinitis after trauma (*n* = 1; histology: chronic rhinitis), and an OND and nasal outlet stenosis (*n* = 1; histology: lymphoplasmacytic rhinitis). Eight (80%; CI_95%_ 49–96) and two (20%; CI_95%_ 4–51) of the 10 dogs were normocephalic and brachycephalic, respectively. Seven and three of the 10 dogs were male [70%; CI_95%_ 40–89; intact (*n* = 3) and neutered (*n* = 4)), and female (30%; CI_95%_ 11–60; intact (*n* = 1) and neutered (*n* = 2)], respectively.

Control dogs. Ten normocephalic dogs (nine beagles and one mixed-breed dog) without clinical signs were included. Histopathologic examination showed normal physiological nasal mucosa. Four and six of the 10 dogs were male (40%; CI_95%_ 17–69; all neutered) and female (60%; CI_95%_ 31–83; all neutered), respectively.

### Cross-group characteristics

Blood count **(**Table 1**).** Of the 55 dogs with nasal disease and 10 control dogs examined, the hematocrit was within the reference range (0.44–0.52 l/l) in 37/65 dogs. It was slightly decreased in 10/65 dogs [< 0.3 l/l in one dog (carcinoma (*n* = 1)]; 0.3–0.4 l/l in 5 dogs [carcinoma (*n* = 1), sarcoma (*n* = 1), and IR (*n* = 3)], and 0.4–0.45 l/l in 4 dogs [carcinoma (*n* = 3), IR (*n* = 1)]. It was slightly above the reference range in 18 dogs [carcinoma (*n* = 4), sarcoma (*n* = 7), benign tumors (*n* = 1), IR (*n* = 3), others (*n* = 2), and CG (*n* = 1)]. The mean platelet count was slightly above the reference range in 6 dogs (3 carcinomas, 2 sarcomas, 1 other) and beneath it in one dog (benign tumor; platelet count < 150 g/l).

Although the leukocyte values of the dogs in all groups, except those with benign tumors, were significantly higher than the values of those in the CG, only dogs with IR and other benign inflammatory rhinitis showed a median leukocyte value slightly above the upper reference range (Table 1). Only four dogs showed leukocyte counts > 20 G/l [carcinoma (*n* = 2), sarcoma (*n* = 1), and IR (*n* = 1)]. Leukopenia was present in only one dog (5.8 G/l) in the CG.

Blood Ratios. NLR, PLR, and AGR did not differ among the subgroups of dogs with ND and nasal pathology (carcinoma, sarcoma, benign tumors, IR, and others). Similarly, except for the PLR, there were no significant differences between benign and malignant NPs.

**Fig. 1:**
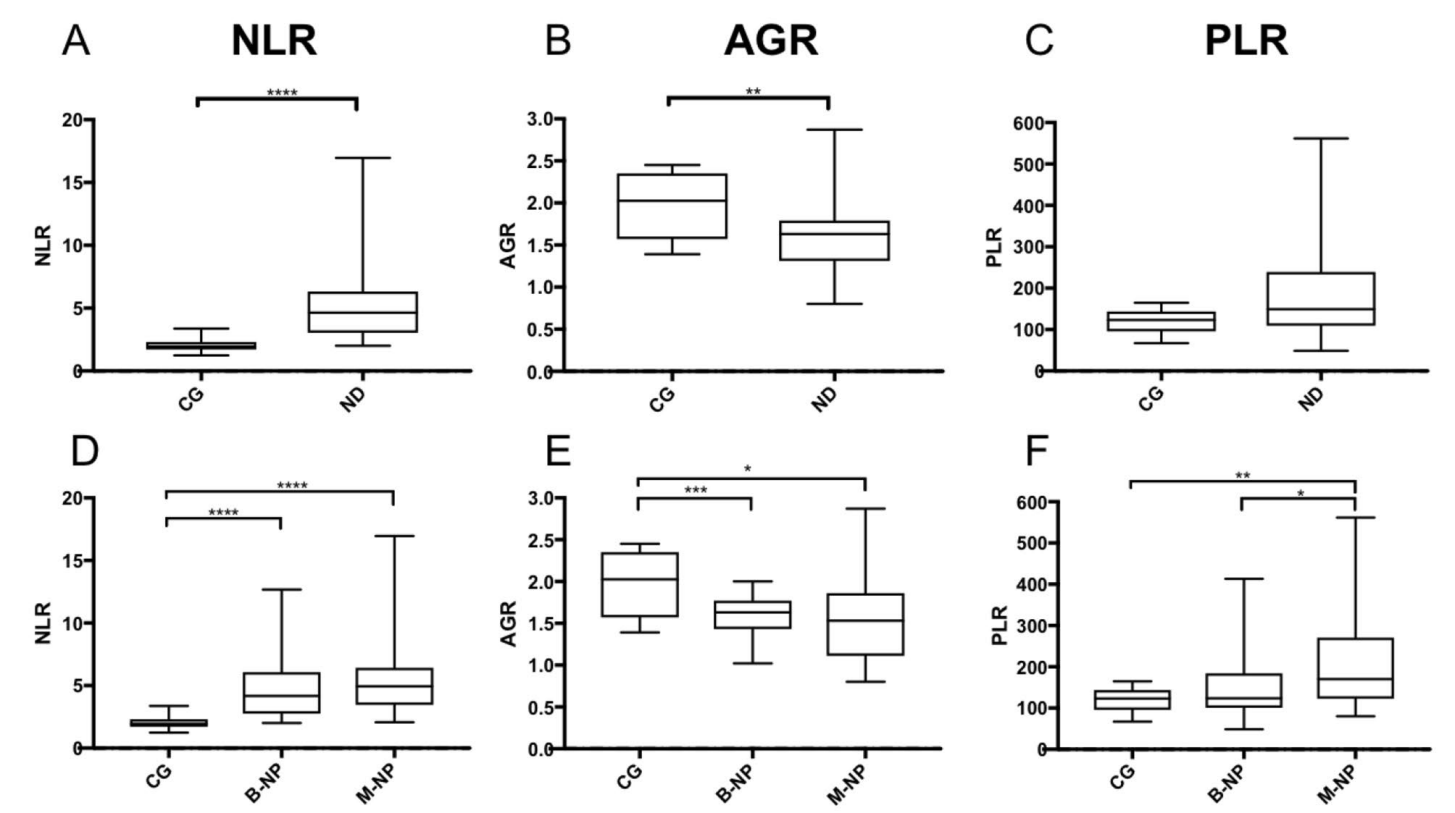
NLR, AGR, and PLR were compared between the control group (CG) and dogs with nasal discharge (ND) in general (*n* = 55, top row, **A–C**); and between the control group (CG, *n* = 10) and dogs with benign (B-NP, *n* = 30) and malignant (M-NP, *n* = 25) nasal cavity pathology (bottom row, **D-F**). The NLR was significantly higher in dogs with ND than in CG (*p* < 0.0001), as well as in dogs with benign or malignant NP (*p* < 0.0001). AGR was significantly lower in dogs with ND compared to the CG (*p* = 0.0025). In contrast to CG, AGR was significantly lower in benign (B-) NP (*p* = 0.0006), and malignant (M-) NP; *p* = 0.0202. PLR was not significantly increased between CG and dogs with ND. However, PLR of dogs with M-NP was significantly higher than that of B-NP (*p* = 0.0244) and that of the CG (*p* = 0.0074). Data are shown in box and whisker plots. Upper and lower boxes represent the 25th and 75th percentiles (lower whiskers = minimum, upper whiskers = maximum values) and the line represents the median

**Fig. 2:**
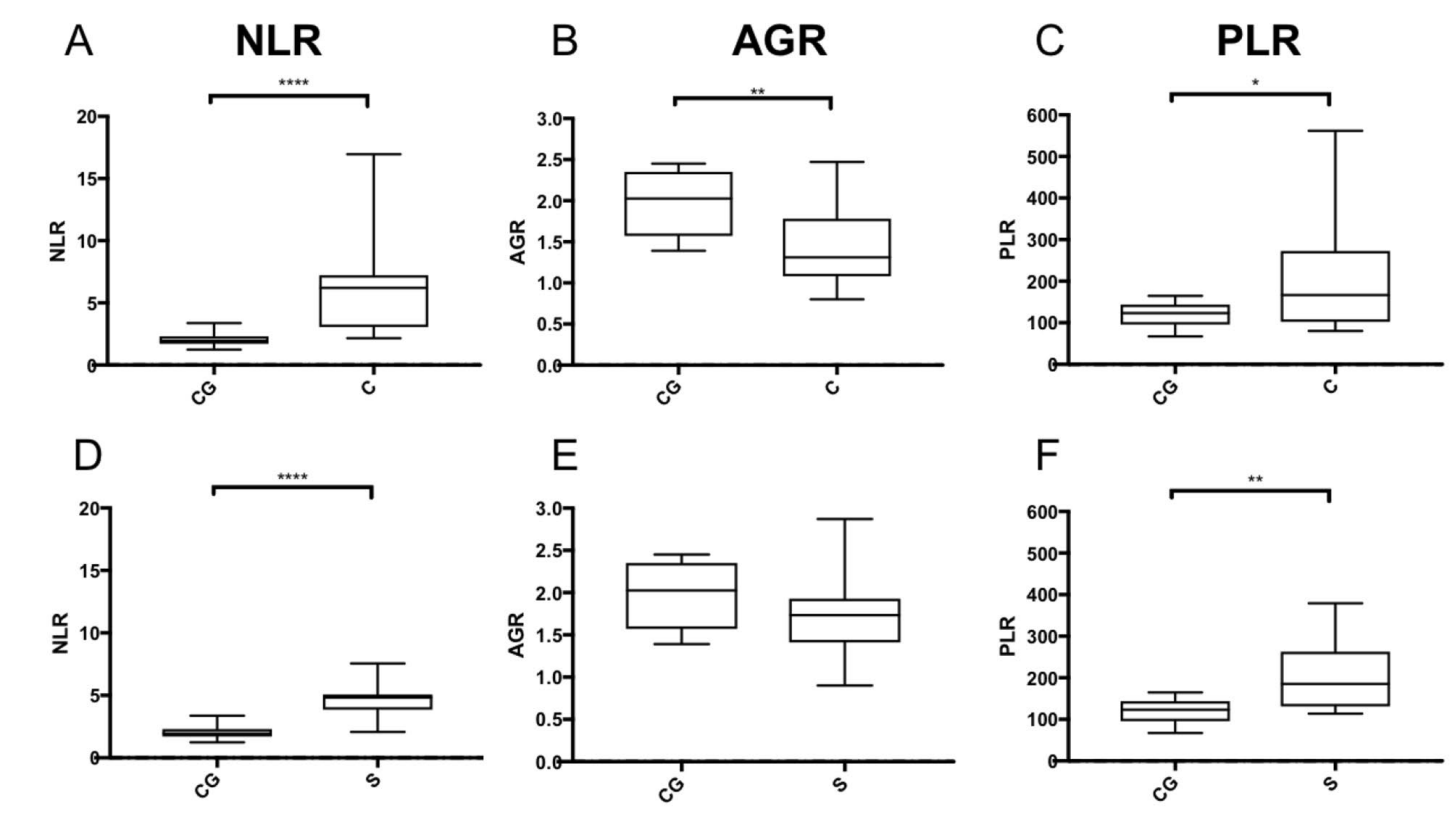
NLR, AGR, and PLR in carcinomas (C, *n* = 13) and sarcomas (S, *n* = 12) of the nasal cavity. **A, D** NLR is comparably significantly increased in dogs with carcinomas (*p* < 0.0001) and sarcomas (*p* < 0.0001) compared to the Control Group (CG, *n* = 10). **B, E** Compared to CG, AGR is significantly decreased only in dogs with carcinomas (*p* = 0.0063). **C, F** PLR, on the other hand, is significantly more increased in dogs with sarcomas (*p* = 0.0071) than in dogs with carcinomas (*p* = 0.0493) compared to the control group. Data are shown in box and whisker plots as described in Fig. 1

The NLR was significantly increased (*p* < 0.0001; Fig. 1) among dogs in the CG (median: 1.95 [IQR: 1.71–2.32]) and those with ND (median: 4.64 [IQR: 3.04–6.32]). In general, the ROC curve analysis to distinguish healthy dogs (CG) from dogs with ND showed an AUC of 0.95 (95% CI: 0.89–1.01) for NPs, an NLR ≥ 2.39 showed a sensitivity of 92.6% and a specificity of 90% to detect NP. However, the NLR did not differ for benign (median: 4.17 [IQR: 2.75–6.07]) and malignant NPs (median: 4.94 [IQR: 3.46–6.42]). The NLR of the benign NPs group (*p* < 0.0001), as well as the NLR of the malignant NPs group (*p* < 0.0001), was significantly increased compared to the CG (Fig. 1). Malignant NPs: The NLR was significantly increased in dogs with carcinomas (*p* < 0.0001) and sarcomas (*p* < 0.0001) compared with dogs in the CG (Fig. 2). Benign NPs: The NLR was significantly increased in all dogs with benign NPs (IR: *p* < 0.0001; other benign inflammatory rhinitis: *p* = 0.0008; and benign NN: *p* = 0.0031) compared with dogs in the CG.

In contrast to the CG with a mean AGR of 1.98 ± 0.39, the AGR was significantly decreased in dogs with ND in general (mean: 1.57 ± 0.38, *p* = 0.0025; Fig. 1), those with malignant (mean: 1.55 ± 0.49, *p* = 0.0202) and benign NPs (mean: 1.59 ± 0.25, *p* = 0.0006). The AGR values of dogs with benign and malignant NPs did not differ. Malignant NPs: While dogs with sarcomas did not show significantly lower AGR than those in the CG (Fig. 2), the AGR of dogs with carcinomas was significantly lower (*p* = 0.0063; Fig. 2). The ROC curve analysis to distinguish healthy dogs from dogs with carcinoma showed an AUC of 0.82 (95% CI: 0.63–1.0). Compared to healthy dogs, for the detection of dogs with nasal carcinoma an AGR < 1.45 showed a sensitivity of 61.5% and a specificity of 90%. Benign NPs: The AGR was significantly decreased in dogs with IR (*p* = 0.0014) and dogs in the “others” group (*p* = 0.0213) compared to that in the CG. The AGR was not significantly lower in dogs with benign NN than in those in the CG.

In the comparison of the PLR between dogs in the CG (median: 123.5 [IQR: 95.84–143.9]) and those with ND (median: 149.3 [IQR: 109.3–239.4]), the PLR was not significantly different (Fig. 1). Although there was no significant difference between dogs in the CG and dogs with benign NPs (IR, others, Benign NN; median: 123.9 [IQR: 100.9–184.1]), the PLR was significantly increased in dogs with malignant NPs (median: 170.4 [IQR: 122.9–271]) compared to those in the CG (*p* = 0.0074; Fig. 1). The ROC curve analysis to distinguish healthy dogs from those with malignant NPs showed an AUC of 0.79 (95% CI: 0.64–0.94) and compared to healthy dogs, for detection of dogs with malignant NPs, a PLR ≥ 112.4 showed a sensitivity of 88% and a specificity of 50%. Furthermore, PLR was significantly increased in dogs with malignant NPs compared to those with benign NPs (*p* = 0.0244). The ROC curve analysis to distinguish dogs with benign nasal cavity tumors from those with malignant nasal cavity tumors showed an AUC of 0.70 (95% CI: 0.48–0.92). Compared to dogs with benign nasal cavity tumors, for detection of malignant NPs, a PLR of > 127.6 showed a sensitivity of 76% and a specificity of 57.1%. In further group comparisons, PLR was significantly increased in dogs with sarcomas (*p* = 0.0071), and those with carcinomas (*p* = 0.0493) compared to dogs in the CG (Fig. 2).

## Discussion

This is the first prospective study in veterinary medicine examining dogs with ND compared to a healthy CG using an intense work-up, including whole-body CT, upper airway endoscopy, biopsies of nasal mucosa or tumor mass, and nasal cavity swabs for bacteriologic and mycological examinations by culture. These methods made it possible to diagnose nasal cavity disease more accurately. In addition to rhinoscopy, nasal swabs and histopathology, CT of the head is necessary to evaluate the nasal cavity, periodontium, paranasal sinuses, and bony structures in diagnosing nasal cavity disease for optimal diagnosis [1, 3, 5]. Furthermore, CT enables the detection of OND and provides indications for fungal diseases of the paranasal sinuses [1, 3, 5].

In addition to the clinical examination of the participating dogs and the standard blood tests, whole-body CT examination was used to exclude the majority of other diseases influencing examined blood parameters. Additionally, dogs with other diseases or who received corticosteroids 14 days prior to the examination were excluded.

Only a few previous studies report blood parameters in single dogs with nasal cavity diseases. They revealed only either slight or no deviations in individual parameters from reference ranges [12]. In contrast to this study, in these dogs, additional diseases were not completely excluded. Prospective studies evaluating the value of blood count as a supportive tool in diagnostics are lacking. Results of this prospective study were in accordance with the previous reports regarding the individual blood parameters. Paraneoplastic syndromes in nasal tumor diseases, such as erythrocytosis or hypercalcemia, which both have been reported in two dogs [24, 25], were not observed in this study.

In contrast to dogs with only nasal disease, dogs with nasal discharge or epistaxis due to systemic diseases such as ehrlichiosis, leishmaniasis, or multiple myeloma often have significant abnormalities on clinical examination and/or blood work, such as lymphadenomegaly, pallor of the mucous membranes, bleeding tendency, pancytopenia, and hyperproteinemia [11]. The pathomechanism of ND in leishmaniasis or epistaxis in all these diseases is not fully understood; however, coagulopathies or hyperviscosity syndrome have been suspected [11]. Considering the increasing numbers of dogs imported from the Mediterranean, systemic diseases should be ruled out by documenting detailed patient history, thorough clinical examination, and basic blood work, especially in cases of pure epistaxis. For dogs with severe abnormalities in blood work (e.g., severe anemia or thrombocytopenia, very low A/G quotient), diagnostics of other diseases should precede diagnostic investigation of possible nasal disease.

Ratios of two blood parameters are less subject to fluctuation than single parameters, making them more suitable investigative parameters [13]. This study investigated for the first time whether the PLR, NLR, or AGR in dogs with NPs differ from each other or the CG and whether they could be helpful in diagnosis in dogs with nasal disease. In malignant tumor diseases in humans, they are reported to be increased (PLR or NLR) or decreased (AGR).

The present study found significantly higher PLRs in dogs with malignant nasal cavity tumors than in dogs with benign NPs and the CG. Further studies are required to clarify whether the PLR level correlates with prognostic significance. While the PLR is used in human medicine as an indicator of prognosis in malignant tumors [20], it has rarely been described in veterinary medicine. Rejec et al. investigated PLR and NLR and found no significant difference in PLR between patients with malignant oropharyngeal tumors [median: 145.31 (IQR: 73–315.3)], patients with periodontitis [224 (IQR: 75–696)], and the CG [290.5 (IQR: 62.13–1051)] [16]. No significant difference (*p* = 0.58) was observed in PLR in dogs with sepsis [median PLR of 214 (IQR: 58.4–457.3)] and in dogs with systemic inflammatory response syndrome (SIRS) [180.2 (IQR: 52–374.7)] [22]. In the present study, the median PLR in dogs with nasal carcinomas and those with nasal sarcomas was 166 (IQR: 102.1–273) and 185.2 (IQR: 131.2–262.7), respectively. This indicates that the PLR values of dogs with malignant nasal diseases in this study were comparable to PLR in dogs with oropharyngeal tumors or SIRS. The reason for increased platelet count values in tumor dogs may be that platelets are involved in the systemic inflammatory response to malignant tumors, as platelet-associated chemokines may modulate inflammation around the tumor as well as tumor angiogenesis [20]. In this study, PLR was increased only in dogs with malignant tumors (sarcomas > carcinomas) and could provide evidence of a malignant event. The cutoff value for malignant NPs compared to healthy dogs was calculated to be a PLR of ≥ 112.4, with a sensitivity of 88% and a specificity of 50%.

NLR and AGR represent the inflammatory responses of an organism [26]. Both purely inflammatory processes and malignant tumors may result in a tumor-associated systemic inflammatory response [16]. The possible causes include tissue hypoxia or local tissue damage [13]. The consequence is an increase in inflammatory cells such as neutrophil granulocytes [13]. Thus, in human medicine, blood neutrophilia can also be detected in patients with tumor diseases [27]. Furthermore, an establishment of the acute phase response can follow, in which positive acute phase proteins such as C-reactive protein are increasingly formed at the expense of the lower formation of the negative acute phase protein, albumin [13]. Lymphocytes suppress the proliferation of tumor cells by different mechanisms, which is why a low number of lymphocytes is considered an inadequate immune defense [20].

In veterinary medicine, NLR has already been studied in dogs with mast cell tumors, lymphoma, oropharyngeal tumors, and sarcomas. In addition, increased NLR has been found in dogs with all these malignancies [13, 16–18]. In the present study, NLR was significantly increased as a marker of systemic inflammatory response in dogs with malignant tumors and all groups with ND compared with dogs in the CG. Consequently, in contrast to the PLR mentioned above, the NLR cannot be used as an indicator of malignant NPs in dogs with ND. While the median NLR of dogs with NPs in this study was 4.64 (IQR: 3.04–6.32) the dogs with oropharyngeal tumors in the study by Rejec et al. had a significantly higher median of 8.59 (IQR: 1.86–30.7).

Studies have shown that AGR is decreased in dogs with malignant soft tissue tumors (sarcomas) [13], and dogs with leishmaniasis [28]. Hence, it can be used to evaluate therapy for this disease [14, 15]. In the present study, AGR was also decreased in both dogs with malignant tumors and IR compared to those in the CG. Given this observation, it can be concluded that these two diseases especially lead to systemic inflammatory reactions. Nevertheless, it must be emphasized that AGR is only slightly decreased in dogs with NPs compared with symptomatic dogs with leishmaniasis. While the median AGR in the present study was 2.03 in dogs in the CG and 1.63 in dogs with NPs, the AGR in symptomatic and asymptomatic dogs with leishmaniasis was 0.42 (SD ±0.15) and 0.8 (SD±0.31), respectively [29].

IR is a common cause of chronic ND in dogs [1, 8], limiting the quality of life of both owners and dogs [30]. Therapy can be difficult; therefore, great owner compliance is required [8]. While mycological examination of nasal mucosa swabs has to be negative, microbiological examinations may be positive because primary bacterial infections of the canine nasal mucosa have not been scientifically proven, and therapy for dogs with IR using conventional antibiotics usually does not result in a cure [1, 8]. This is why dogs with IR were considered a separate group in this study, showing benign rhinitis with ND but without an apparent reason, such as an FB, a tooth root process, or fungal infection. It is important to emphasize that different types of nasal mucosa inflammation can be present in dogs with IR and diagnosis cannot be made solely based on the results of histopathologic examination [3]. The types of mucosal inflammation found in dogs with IR, including lymphoplasmacytic inflammation, can also be observed in dogs with OND or nasal FB [3].

In this study, AGR was decreased in dogs with IR. While polyps are observed in dogs with chronic rhinosinusitis in human medicine [31], and hamartomas are also discussed as a consequence of persistent nasal mucosal inflammation [32], it is striking that AGR was not significantly decreased in dogs with benign tumors in contrast to those with IR (or with malignant tumors or carcinomas). Consequently, the pathomechanisms of canine IR and rhinitis with polyps or hamartomas may differ and require further investigation. Unfortunately, all the examined ratios are not diagnostically helpful in differentiating between benign nasal cavity diseases, such as IR and SNA.

It should be noted that the dogs with ND were recruited at a tertiary institution; therefore, a selection bias cannot be excluded. In general, for the clinical utility of blood ratios, it should be noted that dogs with NPs may also have other diseases that may affect blood ratios. However, the present study largely excluded dogs with these different diseases. Regardless of the strict exclusion criteria, malignant NN was the major cause of ND in this study, accounting for 45% of the cases. Additionally, normocephalic dogs (84%) were significantly more frequently affected than brachycephalic dogs (16%), as already described in the literature [3]. Benign nasal tumors have been rarely observed in the literature [33, 34]. In a comparative study by the authors over 2 years, tumor tissue in the nasal cavity between the turbinates could only be identified as malignant [3], which is why, in addition to the fact that malignant nasal tumors are surrounded by inflammatory tissue, a repeat biopsy is advised and was performed in this study [2].

This study had some limitations. First, is the “others” group, in which dogs with various benign and inflammatory diseases such as OND or SNA were combined due to the low number of dogs. As described in other studies using acute-phase proteins, SNA, which was observed here primarily secondary to a foreign body, could lead to more significant blood changes [35]. Further studies in dogs with SNA are needed to confirm this hypothesis. Second limitation is the CG, which is a relatively homogeneous group, including almost only normocephalic dogs of one breed (beagles).

## Conclusion

Dogs with nasal discharge due to nasal cavity pathology usually do not have marked abnormalities in routine blood tests, even in cases of epistaxis. The combination of an elevated NLR > 2.39, a decreased AGR < 1.45, and an elevated PLR > 112.4 may indicate malignant NPs. Our findings in conjunction with previous evidence indicate that in dogs with nasal discharge, high-grade abnormalities in blood counts, such as hematocrit, leukocytes, and platelets and a marked decrease in AGR to < 0.8, may be indicative of systemic disease [11, 36].

## Methods

### Selection criteria & ethics approval

A prospective blinded study based on an animal experiment application with ethics approval (Germany, Saxony, animal experiment subject to approval TV 02/18) has been described. Dogs presenting with ND to the ear, nose, and throat (ENT) unit of the Clinic for Small Animals at the University of Leipzig between August 2018 and July 2020 were included in the study after written informed consent was obtained from the owners of the dogs that were enrolled in the study.

All examinations were performed in a standardized manner and in accordance with the guidelines and regulations according to TV02/18. Blood samples were collected before anesthesia induction, and the dogs fasted for 12 h. Under anesthesia, a whole-body CT examination with contrast medium was performed, followed by rhinoscopy with bacteriological and mycological examinations of nasal mucosal swabs by culture and histopathological examination of nasal mucosa biopsies or tissues endoscopically classified as tumor tissues.

Dogs diagnosed with other tumor diseases, systemic diseases, or pulmonary pathologies in our examinations (whole-body CT, blood examinations) or those that had been administered corticosteroids within 14 days before presentation to the clinic were excluded. This was because other diseases or corticosteroid administration could affect blood parameters and therefore blood ratios.

Treatment of the different nasal diseases was conducted in majority of the cases by an endonasal endoscopic approach and according to the treatment recommendations for the different nasal diseases. On average, individual patient follow-up through check-ups performed under anesthesia and/or telephone calls was available for up to 2 years after participating in the study.

### Anesthesia

Anesthesia was induced for CT and rhinoscopy by sedating the dogs with medetomidine (5 µg/kg, Domitor^®^, Orion Pharma, Hamburg, Germany) administered intramuscularly and intravenous administration of diazepam (0.5 mg/kg, Rotexmedica, Trittau, Germany), levomethadone (0.25 mg/kg, L-Polamivet^®^, MSD, Haar, Germany), ketamine (2 mg/kg, Ketavet^®^, Pfizer Pharmacia, Berlin, Germany), and propofol dosed according to effect (1–3 mg/kg iv, Narcofol^®^; CP-Pharma, Burgdorf, Germany). All animals were endotracheally intubated, and anesthesia was maintained with isoflurane (2 vol% vaporizer setting; IsoFlo^®^, Essex Tierarznei, Munich, Germany) in 50 vol% oxygen under volume-controlled intermittent positive pressure ventilation. Then a maxillary nerve block (intraoral approach) with a total volume of 0.3–0.9 ml (0.5% bupivacaine (Carbostesin 0.5%^®^, Aspen Germany GmbH, Munich, Germany) and 2% lidocaine (Xylocitin-loc 2%^®^, Mibe GmbH, Brehna, Germany) in a 2:1 ratio) was placed depending on the animal’s size.

### Computed tomography

A six-line spiral CT (Philips Brilliance CT MX 8000 IDT 6, Philips Healthcare, Hamburg, Germany) was used for 31 dogs in this study. All animals were examined in the thoracic-abdominal position. Their heads were positioned in a mostly floating position using a special positioning aid so that the hard palate was parallel to the table. Care was taken to ensure exact lateral symmetry during the positioning. After the planning scan, a native diagnosis of the head and neck was performed. The slice thickness was 1 mm, collimation was 0.75 mm, rotation time was 0.5 s, and increment was 0.5. An automatic dose adjustment and a high-resolution reconstruction filter were used. The generated images were analyzed using a 512 × 512 matrix. Subsequently, the remaining body was examined, and the slice thickness was 2 mm. Native examinations were followed by contrast examinations. The tests were performed using the bolus-tracking method. The measurement volume for the automatic start of the examination was placed in the carotid artery at the level of the caudal end of the head in all dogs. A threshold of 100 HU was chosen; after reaching this value, a contrast examination of the head (slice thickness: 1 mm) and the rest of the body (slice thickness: 2 mm) was performed.

The remaining animals were examined using a 256-line spectral CT scanner (Philips IQon Spectral CT, Philips Healthcare, Hamburg, Germany). The positioning of the animals was identical. The examination parameters were almost identical to those of the device used initially; only the body scan was performed with a 1-mm slice thickness.

CT images of the head were examined for fluid or soft tissue isodense or hypo-dense material in the nasal cavity (mass, secretions, and air), conchal atrophy and osteolytic processes of the bony boundary of the nose, effects on the turbinates, septum, and frontal sinus, dental root disease, and positional changes of the teeth, to establish a diagnosis of nasal pathology.

### Diagnostic rhinoscopy and sinuscopy

The upper airways in all animals, including the nose, nasopharynx, pharynx, larynx, and trachea, were examined according to a standardized endoscopy protocol. Rigid anterograde and retrograde endoscopies without irrigation were performed in the sternal position (HOPKINS® optics, 0°, 2.7 mm, 18 cm, and 0°, 4 mm, 30 cm; HOPKINS® optics 120°, 4 mm, 18 cm; continuous video documentation by Image1 S 3-chip FHD camera head TH100; KARL STORZ, Tuttlingen, Germany). A standardized collection of nasal mucosal swabs and sampling of nasal mucosal biopsies at the ventral turbinate or tumor biopsies were performed under an endoscopic view parallel to the endoscope and not through a guide shaft. A frontal sinus opening was performed via an endonasal approach by interventional endoscopy for frontal sinus filling on CT (exclusion of SNA), followed by an endonasal sinuscopy.

### Laboratory tests

Blood samples were taken from the cephalic vein before anesthesia to prevent the influence of anesthesia on blood tests. Blood count, including differential blood count (EDTA blood, polypropylene tubes with 1.6 mg EDTA/ml blood and platelet and leucocyte values in G/l) and blood chemistry (heparin plasma) tests, and photometry or potentiometry (potassium, sodium; device Cobas c701; Roche) were performed in a blinded fashion at Laboklin (Bad Kissingen, Germany; method flow cytometry (device ADVIA 2120i; Siemens). The NLR, PLR, and AGR were calculated based on the blood count and chemistry test results.

Nasal mucosal swabs were obtained endoscopically from the nasal cavity in a standardized manner [3] and examined microbiologically and mycologically by culture at Idexx Laboratories (Kornwestheim, Germany). A bacteriological examination by culture was considered positive by this third-party laboratory if a specific bacterium was detected with an increased bacterial count. However, because primary bacterial rhinitis has not been described or documented [1], elevated bacterial counts were considered to be either a secondary infection or a reflection of airway colonization [37].

The histopathological examination was performed on hematoxylin-eosin sections in the specialist practice for pathology and by veterinary pathologists (Fachpraxis für Tierpathologie, Dr. W. v. Bomhard (Dipl. ECVP), Munich, Germany). Additionally, Periodic Acid-Schiff (PAS) staining was performed in cases of questionable mycotic infection.

### Grouping

#### Total NPs

All dogs with ND and NPs in this study were included in this group.

#### Malignant NPs

Dogs with a nasal mass between the physiological turbinates on CT and endoscopy were grouped into the malignant tumor group according to the histopathological examination results. This group and its subgroups, including carcinomas and sarcomas, were examined.

#### Benign NPs

This group included all dogs with benign and inflammatory processes in the nasal cavity, such as benign NN, IR, and other causes.

#### Benign nasal tumors (BT)

According to the histopathological examination results, dogs with a benign nasal mass between the physiological turbinates on CT and endoscopy were grouped into the benign tumor group. After initial endonasal endoscopic removal of the benign masses through the nasal entrance, all dogs with benign tumors were reexamined and biopsied endoscopically in a follow-up examination 2 months after the initial examination because malignant tumors are observed more frequently than benign tumors in dogs [1, 3]; hence, there was a possibility of histopathologic misdiagnosis [2]. The diagnosis of a benign process (polyp or hamartoma) was confirmed. In contrast to the diagnosis in human medicine, in which nasal polyps can be part of chronic rhinosinusitis, the etiopathogenesis of hamartomas in the setting of chronic rhinosinusitis in dogs is suspected but not proven. Therefore; dogs with benign nasal mucosal tumors were considered separately. This was performed to compare inflammatory parameters to malignant nasal cavity tumors.

#### IR

Dogs in whom no primary cause of ND could be diagnosed on CT (including the roots of the teeth) and in-depth endoscopy were sorted into the IR group (diagnosis of exclusion). In these dogs, the mycological examination results (fungal culture and histopathology) were negative. In contrast, bacteriological examinations by aerobic culture could be positive in the sense of an increased bacterial count of various bacteria. However, since the existence of primary bacterial rhinitis has not been confirmed in the literature [1], it was evaluated in the sense of a secondary infection or colonization. Since it has been described that dogs with IR may have different histological types of nasal mucosa inflammation [3], and the clinician may accordingly encounter this histologically heterogenic group, no selection was made in this group with respect to the type of inflammation of the nasal mucosa. This group was considered separately as having benign inflammatory NPs because the etiology of IR is not conclusively understood, and allergic and autoimmune processes are also discussed. Furthermore, these dogs are often difficult to adjust therapeutically; therefore, markers that allow monitoring by less invasive means would be helpful in this group.

#### Dogs with another benign inflammatory rhinitis (others, O)

This included all other forms of NPs with rhinitides, such as OND, nasal FB, or fungal diseases. In these dogs, a bacteriological examination by culture could be positive in addition to a positive mycological examination.

#### Control group (CG)

This group included 10 clinically healthy, normocephalic dogs without evidence of ND or NPs on CT examination or endoscopy of the upper respiratory tract. The histopathological examination of nasal mucosal biopsies was unremarkable, and bacteriological and mycological examinations by culture were negative. In addition, the blood status (clinical chemistry and hematology) was unremarkable in these dogs. Similarly, a clinical control examination after 3 months revealed no abnormalities.

### Statistical analyses

Statistical analyses were performed using GraphPad Prism (v7 GraphPad Software, La Jolla, CA, USA). Fractions of totals were calculated and the 95% confidence intervals (CI_95%_) were determined using the Wilson/Brown method. Data were tested for normality using the D’Agostino & Pearson normality test and Shapiro-Wilk Normality test. Normally distributed data were specified as mean ± SD (standard deviation), while non-parametric data were specified as median and IQR (interquartile range). Comparisons among parametric data were made after testing for equality of variance using the Brown-Forsythe test and Bartlett’s test, one-way ANOVA (with Bonferroni multiple comparisons test correction), or by Kruskal-Wallis Test (with Dunn´s multiple comparisons test correction) for non-normally distributed data. The unpaired t-test and Mann-Whitney U test were used for post hoc pairwise comparison of two parametric data and non-parametric data, respectively [35]. Statistical significance was set at *p* < 0.05.

Receiver operating characteristic (ROC) curves were determined to evaluate the sensitivity and specificity for diagnosing NPs (supergroup, subgroup). The ROC curves showed sensitivity versus specificity such that the area under the curve (AUC) varied from 0.5 to 1.0, with higher values indicating increased discrimination.

## Data Availability

The datasets used during the current study are available from the corresponding author on reasonable request.

## Abbreviations

AGR: Albumin-to-globulin ratio
C: Carcinoma
CG: Control group
CI_95%_: 95% Confidence interval
CT: Computed tomography
ENT: Ear, nose, and throat
FB: Foreign bodies
IR: Idiopathic rhinitis
IQR: Interquartile range
ND: Nasal discharge
NLR: Neutrophil-to-lymphocyte ratio
NN: Nasal neoplasms
NP: Nasal cavity pathology
O: Others
OND: Oronasal defects; connections between the oral and nasal cavities, such as cleft palate, periodontopathies, oronasal fistulas
PLR: Platelet-to-lymphocyte ratio
S: Sarcoma
SD: Sarcoma
SNA: Sinonasal aspergillosis
